# Intravesical prostatic protrusion may affect early postoperative continence undergoing robot-assisted radical prostatectomy

**DOI:** 10.1186/s12894-020-00740-0

**Published:** 2020-10-21

**Authors:** Katsuya Hikita, Masashi Honda, Shogo Teraoka, Ryoma Nishikawa, Yuske Kimura, Panagiota Tsounapi, Hideto Iwamoto, Shuichi Morizane, Atsushi Takenaka

**Affiliations:** Division of Urology, Department of Surgery, Faculty of Medicine, Tottori University, 36-1 Nishicho, Yonago, Japan

**Keywords:** Prostate/diagnostic imaging, Prostatectomy/adverse effects, Urinary incontinence/surgery, Robotic surgical procedures/adverse effects, Quality of life

## Abstract

**Background:**

In this study, we investigated the effect of preoperative prostate morphology, especially intravesical prostatic protrusion (IPP), on continence after robot-assisted radical prostatectomy (RARP).

**Methods:**

Retrospective analysis was applied to patients who underwent RARP between October 2010 and July 2014. The following parameters were assessed in all patients: age, body mass index (BMI), prostate-specific antigen, magnetic resonance imaging and pressure-flow studies findings. The impact of preoperative and intraoperative factors on postoperative urinary incontinence (UI) was assessed using multivariate logistic regression analysis. To evaluate the effects of IPP, the patients were divided into groups according to the IPP length: Group 1, < 5 mm and Group 2, ≥ 5 mm. The International Prostate Symptom Score (IPSS), Overactive Bladder Symptom Score, Quality of Life index and the number of pads used were assessed.

**Results:**

A total of 119 patients were eligible for this study. Multivariate analyses showed that IPP (odds ratio (OR) 1.14, 95% confidence interval (CI) 1.02–1.28, *p* < 0.05) and nerve-sparing (NS) (OR 0.23, 95% CI 0.18–0.61, *p* < 0.01) were significant factors related to UI in the first month after RARP. Twelve months after RARP, multivariate analyses revealed that only NS is a factor related to postoperative UI (OR 0.23, 95% CI 0.18–0.61, *p* < 0.01). The comparison of Groups 1 and 2 indicated significant differences in age (*p* < 0.01), prostate volume (*p* < 0.01), total IPSS and voiding symptom score (*p* < 0.05), compliance (*p* < 0.01), and detrusor pressure at maximum flow (*p* < 0.01). Group 1 had a higher continence rate (38.0%) than Group 2 (20.8%) in the first month after RARP (*p* < 0.05), but the difference was no longer significant from the third month after RARP. The total IPSS and voiding symptom scores were significantly different between the two groups before RARP, however, the significant difference disappeared from the first month after RARP.

**Conclusions:**

The data suggest that IPP affects early postoperative UI. Although NS was strongly involved in UI in the early and later stages after RARP, IPP had no effect on UI in the later stages.

## Background

To date there is a variety of treatments for localized prostate cancer (PCa) that can be performed, including active surveillance, radiation therapy and radical prostatectomy. In particular, the robot-assisted laparoscopic radical prostatectomy (RARP) has become a widely performed type of treatment in patients with PCa due to low blood loss and transfusion rates, shorter hospital stay, and fine surgical manipulation [1]. Although RARP is less invasive than conventional open radical prostatectomy (ORP) or laparoscopic radical prostatectomy (LRP), there is postoperative urinary incontinence (UI), as with other procedures. UI after radical prostatectomy has a negative effect on patient’s quality of life (QOL) and has a higher impact in sexual function [2]. So far various surgical techniques have been reported to reduce postoperative UI after RARP [3].

Several studies reported that RARP provides earlier urinary continence compared to ORP and LRP [1]. It has been reported that 17.3% of patients undergoing RARP had urinary continence immediately after catheter removal. The 12-month urinary recovery after RARP ranged from 84 to 97% better than after ORP, where urinary recovery ranged from 60 to 93% and after LRP ranged from 66 to 95% [1, 4].

On the other hand, patient’s factors have also been noted regarding UI after surgery. Several studies have suggested that age, body mass index (BMI), preoperative International Prostate Symptom Score (IPSS) and prostate volume (PV) affect early UI after RARP [1]. Patients with severe lower urinary tract symptoms (LUTS) showed improvement in their subjective symptoms after RARP [5]. Alternatively, reports have investigated the association between benign prostatic hyperplasia (BPH) patterns and early continence after RARP [6]. Currently, several markers have been identified as significant in the clinical progression of BPH. Intravesical prostatic protrusion (IPP) predicts the extent of bladder outlet obstruction in pressure-flow studies (PFS). However, there is limited knowledge on the effects of RARP on LUTS, especially in men with both BPH and PCa. RARP is used to treat both BPH and PCa, and opt for radical surgical removal of the prostate. This study investigates the associations between preoperative BPH patterns, preoperative LUTS and UI after RARP. In particular, the impact of preoperative IPP on preoperative and postoperative UI were examined in detail.

## Methods

### Ethics

This study was conducted at the Division of Urology, Tottori University Hospital, Yonago, Japan. The study was approved by the Tottori University Ethics Committee (No. 2545).

### Patients

Patients who underwent RARP for PCa (stages cT1c–cT3b N0 M0) between October 2010 and July 2014 at our department were included in this study. All patients were signed up after being fully briefed in accordance with the institutional ethics committee. Approved informed consent was obtained and patients were informed that the data would be used anonymously for clinical research purposes. All study data were prospectively collected and retrospectively analysed. The age, BMI, prostate-specific antigen (PSA), Gleason score (GS) and clinical stage were recorded. All patients underwent PFS and magnetic resonance imaging (MRI) before RARP. IPSS, Overactive Bladder Symptom Score (OABSS), QOL index and the number of pads used per day were evaluated before RARP and 1-, 3-, 6-, 9- and 12-months after RARP.

### Surgical procedures

All surgical procedures were performed by six surgeons. The four grades of postero-lateral resection of the prostate were the guide for performing the nerve-sparing (NS) techniques. These included: grade 1, intrafascial dissection; grade 2, interfascial dissection; grade 3, extrafascial dissection and grade 4, wide dissection [7]. The NS grade which was used, was based on the MRI findings and GS from the biopsy. The surgeon decided the NS grade preoperatively and intraoperatively. In this study, NS was defined as at least unilateral NS grade 1 or 2, while non-NS was defined as a NS grade of 3 or 4.

### Definition of continence and patient questionnaire

In this study, continence was defined as using no pads, and was assessed in the physician’s interview with the patient at each outpatient visit before RARP and 1-, 3-, 6-, 9- and 12-months after RARP. Urinary symptom status was analysed using the IPSS, OABSS and QOL index. The IPSS consists of seven questions to assess voiding symptoms (incomplete emptying, intermittency, weak stream and straining to void) and storage symptoms (frequency, urgency and nocturia) [8].

### PFS

PFS was performed by a single examiner using the standard method prescribed by the International Continence Society, using a Solar Gold urodynamic system (MMS USA Inc., Dover, NH, USA) [9]. An 8-Fr double lumen catheter was inserted into bladder and normal saline was injected at 50 ml/min. A balloon catheter was placed into the rectum and abdominal pressure was measured. Detrusor pressure was defined as the intravesical pressure minus abdominal pressure. The first desire to void (FDV), maximum cystometric capacity (MCC), bladder compliance, detrusor pressure at maximum flow (PdetQmax) and presence of detrusor overactivity (DO) were recorded.

### MRI measurements

**Fig. 1:**
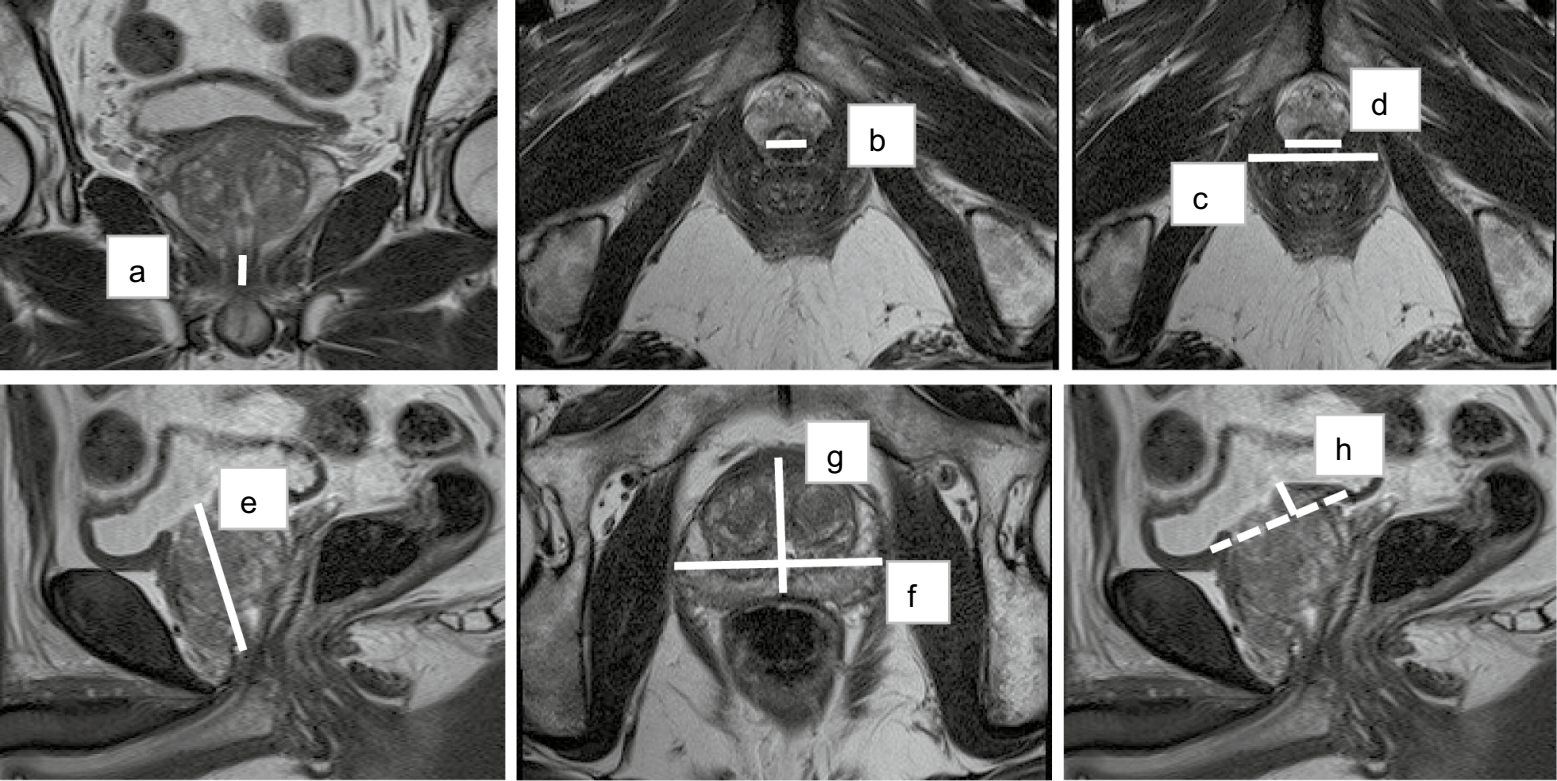
MRI measurements. *a* is the MUL. MUL was measured as the distance of the apex of the prostate to the base of the bulbous urethra. *b* is the MUW. MUW was defined as the maximum diameter of the membranous urethra. LT was calculated as (*c* − *d*)/2. *c* stands for the outer levator distance and *d* stands for the inner levator distance. PV was calculated as *e* × *f* × *g* × π/6/1000. *e* is the maximum height of the prostate, *f* is the maximal prostate width and *g* is the maximal prostate length. Intravesical prostatic protrusion [*h* (mm)], which is measured from the tip of the protruding prostate to the base of the bladder (mid-sagittal T2-weighted image)

The PV, IPP, membranous urethra length (MUL), membranous urethra width (MUW) and levator thickness (LT) were measured using MRI. The PV was calculated as height × width × length × π/6 (axial and mid-sagittal T2-weighted image). The IPP was measured from the tip of the protruding prostate to the base of the bladder (mid-sagittal T2-weighted image). The MUL was measured as the distance from the prostatic apex to the level of the urethra at the penile bulb on coronal T2-weighted image. The MUW was defined as the maximum diameter of the membranous urethra (axial T2-weighted images). LT was calculated as (the outer levator distance minus the inner levator distance)/2 on axial T2-weighted image (Fig. 1).

### The effect of IPP

The effect of IPP was investigated by stratifying the IPP into two levels. The patients were subsequently categorised into two groups based on the IPP length: Group 1 (< 5 mm) and Group 2 (≥ 5 mm). To confirm the difference between the two groups, we compared the pad-free rate, IPSS, OABSS and QOL index before RARP and at 1-, 3-, 6-, 9- and 12-months after RARP.

### Statistical analysis

The data were presented as median values and interquartile range (IQR) and evaluated using the Mann–Whitney U test and Yates chi-square test, where a p -value of less than 0.05 was considered significant. Logistic regression analysis was performed to determine independent predictive values of the main risk factors of UI that have been reported in previous studies (age, BMI, PV, IPSS, OABSS, bladder compliance, NS, MUL, LT and IPP) in the first month and 12 months after RARP [10–14]. The predictors were investigated using multivariate analysis to determine which ones were affected by early and long-term UI. The 95% confidence interval (CI) was calculated for each odds ratio (OR). A p-value of less than 0.05 was considered significant. Statistical analysis was performed using the Statistical Package for the Social Sciences version 19 (IBM, Armonk, NY) software package.

## Results

A total of 119 patients were eligible for this study. The number of patients who did not use pads at 1-, 3-, 6-, 9- and 12-months after RARP were 37 (31.0%), 63 (52.9%), 82 (68.9%), 85 (71.4%) and 91 (76.5%), respectively.

The median values of patient age, BMI and PSA before RARP were 66 (48–76) years, 23.5 (18.0–30.6) kg/m^2^ and 7.8 (3.2–37.1) ng/mL, respectively. The MRI evaluation revealed that the median values of PV, MUL, MUW, LT and IPP before RARP were 26.0 (9.6–66.1) mL, 12.1 (8.9–16.1) mm, 10.6 (9.8–13.5) mm, 11.1 (7.9–15.1) mm, and 3.8 (0.0–16.5) mm, respectively. In terms of the clinical stage, 108 patients had ≤ T2c, 10 patients had T3a and one patient had T3b disease. Twenty-one patients had a GS of 6, 50 patients had a score of 7 and 48 patients had scores ≥ 8. According to NCCN risk stratification, 14 patients were considered at low risk, 53 patients were at intermediate risk and 52 patients were at high risk. A NS procedure was performed bilaterally or unilaterally in 51 patients. Lymph node dissection was performed in 105 patients.

The median total IPSS score, IPSS voiding symptom score, storage symptom score, OABSS and QOL index were 6 (0–28), 4 (0–18), 4 (0–12), 3 (0–10) and 3 (0–6), respectively. The median compliance, FDV, MCC and PdetQmax were 49.8 (5.0–290.2) ml/cmH_2_O, 144 (47–400) ml, 277 (55–470) ml and 45 (6–90) cmH_2_O, respectively. DO was identified in 10 patients (Table 1).

**Table 1:** Patients’ characteristics

	Overall population	Group 1	Group 2	*p* value
Number of patients	119	71	48	
Median age, years (IQR)	66 (48–76)	64 (48–76)	69 (53–76)	< 0.05
Median BMI, kg/m^2^ (IQR)	23.5 (18.0–30.6)	23.6 (18.1–28.9)	22.9 (18.0–30.6)	0.8
Median PSA, ng/mL (IQR)	7.8 (3.2–37.1)	7.8 (3.2–34.6)	7.7 (4.3–37.1)	0.45
*Clinical stage*				
< T2c	108	64	44	0.96
T3a	10	6	4	
T3b	1	1	0	
T4	0	0	0	
*GS of biopsy*				
< 6	21	13	8	0.46
7	50	25	25	
> 8	48	33	15	
*Risk class (NCCN)*				
Low	14	9	5	0.54
Intermediate	53	27	26	
High	52	35	17	
Lymph node dissection (%)	105 (88.2)	62 (87.3)	43 (89.6)	0.75
Nerve sparing (%)	51 (42.8)	28 (39.4)	23 (47.9)	0.15
Median PV, mL (IQR)	26.0 (9.6–66.1)	24.8 (11.1–53.2)	32.8 (9.6–66.1)	< 0.01
Median MUL, mm (IQR)	12.1 (8.9–16.1)	12.1 (9.1–15.3)	12.9 (8.9–16.1)	0.53
Median MUW, mm (IQR)	10.6 (9.8–13.5)	10.8 (9.9–13.4)	10.3 (9.8–13.5)	0.76
Median LT, mm (IQR)	11.1 (7.9–15.1)	11.0 (8.3–15.1)	11.3 (7.9–15.1)	0.66
Median IPP, mm (IQR)	3.8 (0.0–16.5)	1.0 (0.0–4.9)	7.8 (6.2–16.5)	< 0.01
Median IPSS Total score (IQR)	6 (0–28)	6 (0–22)	8 (0–28)	< 0.05
Median IPSS voiding symptom score (IQR)	4 (0–18)	3 (0–15)	5 (0–18)	< 0.01
Median IPSS storage symptom score (IQR)	4 (0–12)	3 (0–12)	4 (0–12)	0.55
Median OABSS score (IQR)	3 (0–10)	2 (0–10)	4 (2–10)	0.45
Median QOL index (IQR)	3 (0–6)	3 (0–6)	3 (0–6)	0.35
Median compliance, mL/cmH_2_O (IQR)	49.8 (5.0–290.2)	55.9 (5.0–290.2)	42.4 (5.4–153.9)	< 0.01
Median FDV, mL (IQR)	144 (47–400)	144 (81–400)	134 (47–301)	0.62
Median MCC, mL (IQR)	277 (55–470)	282 (120–470)	264 (55–431)	0.96
Median PdetQmax, cmH_2_O (IQR)	45 (6–90)	41 (6–89)	48 (28–90)	< 0.01
DO (%)	10 (8.4)	7 (9.8)	3 (6.2)	0.22

Multivariate analysis was performed using the risk factors reported in previous studies (age, BMI, PV, IPSS, OABSS, compliance, NS, MUL, LT and IPP) as being linked to UI in the first month and 12 months after RARP, showed that the IPP (OR 1.14, 95% CI 1.02–1.28, *p* < 0.05) and NS (OR 0.23, 95% CI 0.18–0.61, *p* < 0.01) remained as significant factors in the first month after RARP. At 12 months after RARP, the only factor that remained significantly associated with UI on multivariate analysis was NS (OR 0.88, 95% CI 0.78–0.99, *p* < 0.05) (Table 2).

**Table 2:** Multivariate analyses to identify significant predictors of postoperative urinary incontinence at 1 and 12 months in patients undergoing RARP

Predictors	1 month after RARP			12 months after RARP		
	OR	95% CI	*p*	OR	95% CI	*p*
Age, years	1.04	0.97–1.12	0.23	1.03	0.95–1.12	0.43
BMI, kg/m^2^	0.89	0.75–1.01	0.23	0.97	0.81–1.16	0.72
Nerve sparing, yes/no	0.23	0.18–0.61	0.003	0.88	0.78–0.99	0.03
PV, mL	1	0.96–1.05	0.96	1	0.96–1.05	0.9
MUL, mm	0.91	0.82–1.00	0.06	0.95	0.85–1.10	0.39
LT, mm	1.05	0.92–1.20	0.45	0.98	0.87–1.10	0.74
IPP, mm	1.14	1.02–1.28	0.02	0.99	0.87–1.14	0.96
IPSS Total score	0.95	0.82–1.06	0.33	1.04	0.87–1.14	0.39
OABSS score	1.07	0.79–1.44	0.65	1.06	0.82–1.37	0.66
Compliance, mL/cmH_2_O	0.99	0.99–1.01	0.65	1.01	0.99–1.02	0.2

The patients were stratified into two groups according to IPP length: Group 1 (< 5 mm: n = 71) and Group 2 (≥ 5 mm: n = 48). The comparison of the two groups indicated significant differences in age (*p* < 0.01), PV (*p* < 0.01), total preoperative IPSS score (*p* < 0.05), preoperative IPSS voiding symptom score (*p* < 0.01), bladder compliance (*p* < 0.01) and PdetQmax (*p* < 0.01) (Table 1).

**Fig. 2:**
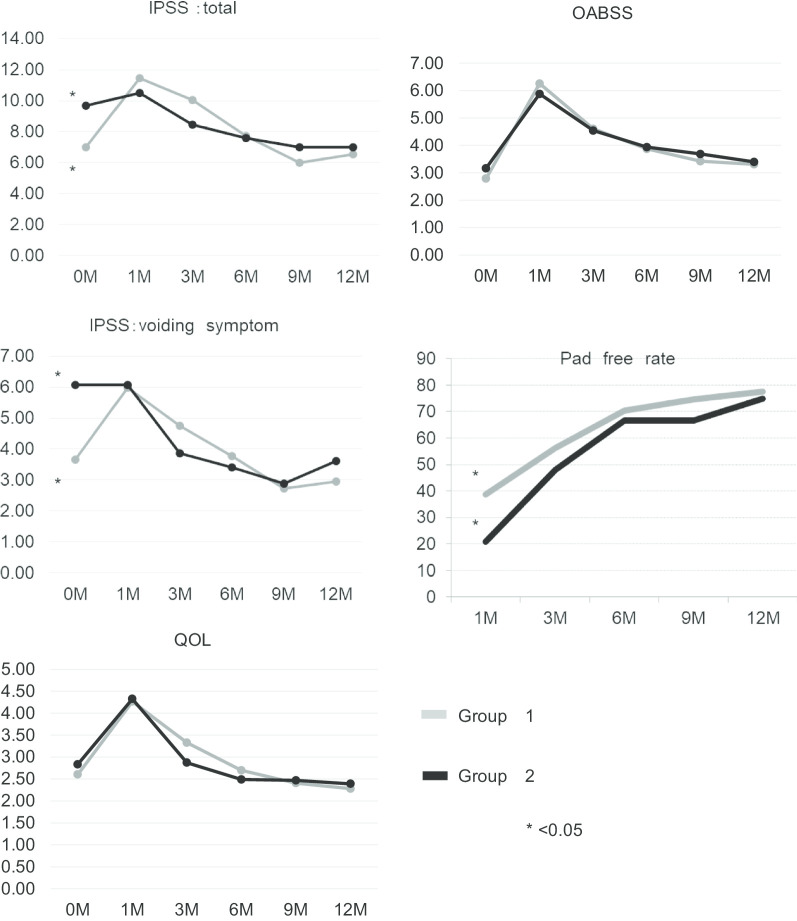
The distribution of the total International Prostate Symptom Score (IPSS), voiding symptom score, QOL index, OABSS score, and pad-free rate between groups 1 and 2 preoperatively and at 1-, 3-, 6-, 9-, and 12-month follow-ups

With respect to the total IPSS and voiding symptom score, a significant difference was observed between the two groups before RARP (*p* < 0.01), but the significant difference disappeared from the first month after RARP. The IPSS storage symptom score, QOL index and OABSS were not significantly different between groups 1 and 2 both before and after RARP (Fig. 2).

The evaluation of UI in Group 1 showed that the number of patients who did not use pads at 1-, 3-, 6-, 9- and 12-months after RARP were 27 (38.0%), 40 (56.3%), 50 (70.4%), 53 (74.6%) and 55 (77.5%), respectively. In Group 2, the number of patients who did not use pads at 1-, 3-, 6-, 9- and 12-months after RARP were 10 (20.8%), 23 (47.9%), 32 (66.7%), 32 (66.7%) and 36 (75.0%), respectively. Group 1 had a higher continence rate than Group 2 in the first month after RARP (p < 0.05); however, the significant difference disappeared 3 months onward after RARP (Fig. 2).

## Discussion

The post-RARP UI is influenced by the preoperative patient’s characteristics as well as the surgical technique. In a systematic review, the 12-month UI rates ranged from 4 to 31% with a mean value of 16% after RARP [1]. In our study, the number of patients who did not use pads at 1-, 3-, 6-, 9- and 12-months after RARP were 37 (31.0%), 63 (52.9%), 82 (68.9%), 85 (71.4%) and 91 (76.4%), respectively. The incontinence rate in this study was higher than in the previous reports. One possible reason for the high UI rate in this study was the older age of patients than in the previous reports [1, 15]. It might also be related to the surgical technique used. Studies that evaluated the impact of different surgical techniques on UI after RARP found that posterior musculofascial reconstruction with or without anterior reconstruction was associated with a small advantage in the recovery from urinary continence 1 month after RARP [1, 16]. In our study, both anterior and posterior reconstruction was performed in all cases. Therefore, the effects of the reconstruction could not be assessed. Although bladder neck preservation was not evaluated in this study, it was previously reported to be associated with a higher continence rate after RARP [12, 13]. With respect to NS, this procedure has been known to favour continence. Retrospective studies showed that better continence rates at 9–12 months after LRP or RARP were achieved in patients with at least one completely spared neurovascular bundle [11]. In our study, NS is a solid factor associated with a higher preserved continence rate from the early to the long-term postoperative period. NS was performed in only 51 cases (42.9%), which might have contributed to the high rate of UI observed in this study [16].

Besides the surgical technique, several other factors related to UI after RARP have been mentioned. Some reports show that patient age, BMI, MUL, LT, IPP, race, OABSS and severity of preoperative IPSS score are factors associated with the continence rate after RARP [5, 10–12, 19, 20]. Therefore, we performed multivariate analysis based on these factors in this study. NS and IPP were significant factors for UI during the first month post-RARP. As for NS, as reported by Suardi et al., this factor has an early effect on UI rates and is still a significant factor 12 months postoperatively [16]. However, although IPP affected early postoperative UI, this effect disappeared at 12 months postoperatively.

In this study, we focused on and examined the impact of IPP on UI after RARP. In a previous study, the impact of IPP on the effects of the therapy for BPH was extensively evaluated [21]. RARP provides treatment for both BPH and PCa; opt for radical surgical removal of the prostate. Therefore, the effect of IPP on preoperative urinary status also affects the urinary status after RARP. Additionally, as Grivas et al. reported that IPP predicts the extent of bladder outlet obstruction seen in PFS, it is understandable that IPP affects both preoperative and post-RARP urinary states [6]. However, Grivas et al. also reported that IPP was not a significant factor affecting the continence rate, although their study evaluated outcomes at only 6 and 12 months after RARP [6]. Alternatively, this study is more detailed with evaluations before RARP and at 1-, 3-, 6-, 9- and 12-months after RARP. In this study, IPP was not a significant factor related to the continence rate 12 months after RARP, but was only linked to UI at the first month after surgery. Furthermore, previous reports have also shown that IPP affects UI, with significant differences in the recovery of continence at 3-, 6- and 12-months after surgery [15]. Our study showed that postoperative UI was affected by preoperative IPP at an earlier stage.

Besides the impact of IPP on early post-RARP UI, there were also significant differences between the two groups in bladder compliance, PdetQmax, IPSS total score, IPSS voiding symptom score and PV. A higher grade of IPP would result in a higher degree of subclinical bladder dysfunction before RARP, resulting in a lower rate of urinary continence after RARP. Yamada et al. reviewed 272 patients after RARP. Multivariable analysis showed that the presence of an unstable bladder preoperatively was an independent negative predictor of the recovery of continence within 12 months after surgery [13]. In our study, although IPP affected bladder compliance, there were no significant differences in FDV, MCC, OABSS and IPSS storage symptom score between the two groups. Thus, although IPP potentially have an impact on urinary storage symptoms, no significant difference was found in this study because there were only few cases with a high IPP grade. Hence, different results might have been obtained if the number of cases was higher.

In this study, the total IPSS score and voiding symptom score were preoperatively significantly different between the two groups. However, there was no significant difference after surgery. Therefore, patients with severe preoperative voiding symptoms benefit from RARP.

The limitations of this study were the small number of cases, few cases of severe IPP and lack of evaluation of bladder neck preservation. Since it was difficult to perform bladder neck preservation in high-grade IPP patients, the assessment of bladder neck preservation in cases of IPP facilitates the evaluation of its effect on UI in the future.

Thus, future studies assessing the effect of IPP on UI should include a larger number of severe cases of IPP to enable more detailed study.

## Conclusions

The results of this study suggest that IPP affects urinary continence in the early postoperative period. Although NS was strongly involved in urinary continence in both early and later stages after RARP, IPP had no effect in the later stages. Various preoperative factors are involved in continence after RARP, and IPP is one of those factors. More detailed evaluation of a larger number of cases in the future will help shed more light on this topic.

## Data Availability

The datasets used and/or analysed during the current study available from the corresponding author on reasonable request.

## Abbreviations

PCa: Prostate cancer
RARP: Robot-assisted radical prostatectomy
ORP: Open radical prostatectomy
UI: Urinary incontinence
QOL: Quality of life
LRP: Laparoscopic radical prostatectomy
BMI: Body mass index
IPSS: International Prostate Symptom Score
PV: Prostate volume
LUTS: Lower urinary tract symptoms
BPH: Benign prostatic hyperplasia
IPP: Intravesical prostatic protrusion
PFS: Pressure-flow study
PSA: Prostate-specific antigen
GS: Gleason score
MRI: Magnetic resonance imaging
OABSS: Overactive Bladder Symptom Score
NS: Nerve-sparing
DO: Detrusor overactivity
FDV: First desire to void
MCC: Maximum cystometric capacity
Pdet Qmax: Detrusor pressure at peak urinary flow rate
MUL: Length of the membranous urethra
MUW: Width of the membranous urethra
LT: Levator thickness
IQR: Interquartile range
CI: Confidence interval
OR: Odds ratios
